# Virtual Lifestyle Modification/Brain Health Group for Patients with early AD on DMT and their Support Partners

**DOI:** 10.1002/alz70858_107439

**Published:** 2025-12-25

**Authors:** Katelyn Bourgea, Gretchen Reynolds, Katherine Crocker, Kim Willment

**Affiliations:** ^1^ Mass General Brigham, Boston, MA, USA; ^2^ College of the Holy Cross, Worcester, MA, USA

## Abstract

**Background:**

Modifiable lifestyle factors can significantly impact the onset and trajectory of dementia (Ornish et al., 2024). Without structured support, initiation and maintenance of behavior change remains challenging, particularly for those with mild cognitive impairment (MCI). With the advent of disease modifying therapies (DMT) for Alzheimer's disease (AD), support for healthy lifestyle change is crucial to potentially maximize therapeutic benefit and provide emotional support. Social environmental models of health behavior change suggest that engaging in behavior modification with a support partner (eg, Chandler et al., 2019) may positively influence habit formation (eg, ripple effect; Gorin et al., 2018) yet, many MCI lifestyle interventions neglect to include support partners (e.g., Ngandu et al., 2015), overlooking an opportunity to enhance interventions for patients and support care partners.

**Program Design/Aims:**

This novel Brain Health program is a virtual clinical group for patients with early AD receiving DMT (eg, lecanemab) and their chosen support partners. Duration is 6 months, with sessions held every other week for 60‐90 minutes, led by neuropsychologists. Each month begins with a didactic session, covering psychoeducation on key brain health topics specific to early AD (i.e., cognitive and social engagement, exercise, diet, mindfulness, sleep). Subsequent sessions each month will focus on dyadic check‐ins to develop SMART goals related to lifestyle modification. Questionnaire data will be collected for patients and partners at baseline, 3‐month midpoint, and treatment completion, and at 6‐month and 12‐month follow‐up post‐treatment to assess maintenance of gains. Measures assess various topics including knowledge of dementia risks and lifestyle recommendations, motivation, self‐efficacy, and wellbeing. The primary aim is to provide a supportive clinical service focused on lifestyle modification to optimize brain health, agency, and emotional well‐being for patients receiving DMT for AD and support partners. Secondary aims, grounded in theories of Self‐Determination, motivation, and behavior change, include evaluating mechanisms of change and partner support styles to enhance understanding of effective interpersonal support approaches and barriers/facilitators to lifestyle modification for brain health. Impact will be maximized by implementing a 6‐month group with patients and partners to support lasting behavior change, potentially enhance DMT outcomes, and target multiple levels of prevention.

